# Supplementary material for: Transcriptome Analysis Reveals Vimentin-Induced Disruption of Cell–Cell Associations Augments Breast Cancer Cell Migration
Source: Cells. 2022 Dec 13;11(24):4035. doi: 10.3390/cells11244035 (PMC9776984; doi:10.3390/cells11244035)
Supplement: Supplementary file 1 [file cells-11-04035-s001.zip › DEGs and enrichment analysis.zip!/DEGs and enrichment analysis/WT_VIMvsVector KEGG/GSEA/WT_VIM/CHOLESTEROL_METABOLISM/CHOLESTEROL_METABOLISM(HSA04979).html]

Details for gene set CHOLESTEROL\_METABOLISM(HSA04979)[GSEA]

|  || Dataset | fpkm.sample |
| Phenotype | sample.cls |
| Upregulated in class | WT\_VIM |
| GeneSet | CHOLESTEROL\_METABOLISM(HSA04979) |
| Enrichment Score (ES) | 0.66701984 |
| Normalized Enrichment Score (NES) | 1.1463274 |
| Nominal p-value | 0.0 |
| FDR q-value | 0.28768355 |
| FWER p-Value | 0.498 |
Table: GSEA Results Summary

  

Fig 1: Enrichment plot: CHOLESTEROL\_METABOLISM(HSA04979)      
 Profile of the Running ES Score & Positions of GeneSet Members on the Rank Ordered List

  

| PROBE | DESCRIPTION (from dataset) | GENE SYMBOL | GENE\_TITLE | RANK IN GENE LIST | RANK METRIC SCORE | RUNNING ES | CORE ENRICHMENT || 1 | ENSG00000110245 | APOC3 |  |  | 1154 | Infinity | -0.0195 | Yes |
| 2 | ENSG00000084674 | APOB |  |  | 1781 | Infinity | -0.0300 | Yes |
| 3 | ENSG00000228838 | AL355483.2 |  |  | 2233 | Infinity | -0.0375 | Yes |
| 4 | ENSG00000167780 | SOAT2 |  |  | 2393 | 79.997 | 0.5780 | Yes |
| 5 | ENSG00000081479 | LRP2 |  |  | 3580 | 3.526 | 0.5851 | Yes |
| 6 | ENSG00000231341 | VDAC1P6 |  |  | 3996 | 3.026 | 0.6015 | Yes |
| 7 | ENSG00000100979 | PLTP |  |  | 4511 | 2.643 | 0.6132 | Yes |
| 8 | ENSG00000130203 | APOE |  |  | 4988 | 2.409 | 0.6238 | Yes |
| 9 | ENSG00000130164 | LDLR |  |  | 5405 | 2.231 | 0.6340 | Yes |
| 10 | ENSG00000100300 | TSPO |  |  | 5435 | 2.220 | 0.6506 | Yes |
| 11 | ENSG00000213856 | VDAC1P2 |  |  | 5513 | 2.194 | 0.6663 | Yes |
| 12 | ENSG00000123384 | LRP1 |  |  | 7159 | 1.782 | 0.6522 | Yes |
| 13 | ENSG00000021762 | OSBPL5 |  |  | 7707 | 1.676 | 0.6559 | Yes |
| 14 | ENSG00000172974 | AC007318.1 |  |  | 8506 | 1.546 | 0.6543 | Yes |
| 15 | ENSG00000254595 | AC084337.1 |  |  | 9371 | 1.434 | 0.6508 | Yes |
| 16 | ENSG00000163956 | LRPAP1 |  |  | 9441 | 1.424 | 0.6606 | Yes |
| 17 | ENSG00000131748 | STARD3 |  |  | 9757 | 1.388 | 0.6660 | Yes |
| 18 | ENSG00000078668 | VDAC3 |  |  | 10970 | 1.268 | 0.6553 | Yes |
| 19 | ENSG00000130173 | ANGPTL8 |  |  | 11613 | 1.218 | 0.6538 | Yes |
| 20 | ENSG00000119655 | NPC2 |  |  | 12088 | 1.174 | 0.6549 | Yes |
| 21 | ENSG00000073060 | SCARB1 |  |  | 12368 | 1.151 | 0.6590 | Yes |
| 22 | ENSG00000165637 | VDAC2 |  |  | 12421 | 1.147 | 0.6670 | Yes |
| 23 | ENSG00000073905 | VDAC1P1 |  |  | 14076 | 1.020 | 0.6469 | No |
| 24 | ENSG00000157978 | LDLRAP1 |  |  | 14321 | 1.001 | 0.6505 | No |
| 25 | ENSG00000101558 | VAPA |  |  | 14753 | 0.986 | 0.6508 | No |
| 26 | ENSG00000124164 | VAPB |  |  | 15099 | 0.964 | 0.6524 | No |
| 27 | ENSG00000213585 | VDAC1 |  |  | 15554 | 0.936 | 0.6520 | No |
| 28 | ENSG00000167910 | CYP7A1 |  |  | 15732 | 0.929 | 0.6562 | No |
| 29 | ENSG00000135218 | CD36 |  |  | 16528 | 0.886 | 0.6495 | No |
| 30 | ENSG00000130208 | APOC1 |  |  | 17191 | 0.845 | 0.6449 | No |
| 31 | ENSG00000144959 | NCEH1 |  |  | 17787 | 0.811 | 0.6411 | No |
| 32 | ENSG00000141458 | NPC1 |  |  | 17835 | 0.808 | 0.6465 | No |
| 33 | ENSG00000213398 | LCAT |  |  | 19565 | 0.712 | 0.6227 | No |
| 34 | ENSG00000057252 | SOAT1 |  |  | 19589 | 0.710 | 0.6278 | No |
| 35 | ENSG00000134243 | SORT1 |  |  | 20832 | 0.644 | 0.6118 | No |
| 36 | ENSG00000229036 | VDAC1P8 |  |  | 20873 | 0.642 | 0.6161 | No |
| 37 | ENSG00000007944 | MYLIP |  |  | 21265 | 0.622 | 0.6142 | No |
| 38 | ENSG00000107798 | LIPA |  |  | 22617 | 0.536 | 0.5955 | No |
| 39 | ENSG00000165029 | ABCA1 |  |  | 23537 | 0.475 | 0.5836 | No |
| 40 | ENSG00000118137 | APOA1 |  |  | 23572 | 0.472 | 0.5867 | No |
| 41 | ENSG00000166035 | LIPC |  |  | 24108 | 0.429 | 0.5810 | No |
| 42 | ENSG00000213071 | LPAL2 |  |  | 25307 | 0.322 | 0.5632 | No |
| 43 | ENSG00000167772 | ANGPTL4 |  |  | 25459 | 0.310 | 0.5630 | No |
| 44 | ENSG00000135929 | CYP27A1 |  |  | 25765 | 0.275 | 0.5600 | No |
| 45 | ENSG00000147465 | STAR |  |  | 26443 | 0.196 | 0.5500 | No |
| 46 | ENSG00000198670 | LPA |  |  | 27148 | 0.074 | 0.5387 | No |
| 47 | ENSG00000101670 | LIPG |  |  | 27220 | 0.040 | 0.5378 | No |
| 48 | ENSG00000169174 | PCSK9 |  |  | 27537 | 0.000 | 0.5324 | No |
| 49 | ENSG00000226671 | AC005008.1 |  |  | 27706 | 0.000 | 0.5296 | No |
| 50 | ENSG00000143921 | ABCG8 |  |  | 27843 | 0.000 | 0.5273 | No |
| 51 | ENSG00000110244 | APOA4 |  |  | 29009 | 0.000 | 0.5075 | No |
| 52 | ENSG00000237679 | VDAC1P11 |  |  | 29223 | 0.000 | 0.5039 | No |
| 53 | ENSG00000234702 | VDAC1P3 |  |  | 30677 | 0.000 | 0.4793 | No |
| 54 | ENSG00000087237 | CETP |  |  | 30744 | 0.000 | 0.4782 | No |
| 55 | ENSG00000227254 | VDAC1P12 |  |  | 31124 | NaN | 0.4719 | No |
| 56 | ENSG00000138075 | ABCG5 |  |  | 32131 | NaN | 0.4549 | No |
| 57 | ENSG00000271198 | VDAC3P1 |  |  | 33378 | NaN | 0.4339 | No |
| 58 | ENSG00000250458 | AC093767.1 |  |  | 33587 | NaN | 0.4304 | No |
| 59 | ENSG00000091583 | APOH |  |  | 35951 | NaN | 0.3905 | No |
| 60 | ENSG00000285513 | AP006216.3 |  |  | 36066 | NaN | 0.3887 | No |
| 61 | ENSG00000214855 | APOC1P1 |  |  | 37913 | NaN | 0.3575 | No |
| 62 | ENSG00000232526 | VDAC1P13 |  |  | 38213 | NaN | 0.3525 | No |
| 63 | ENSG00000232809 | VDAC1P10 |  |  | 38847 | NaN | 0.3418 | No |
| 64 | ENSG00000175445 | LPL |  |  | 38877 | NaN | 0.3414 | No |
| 65 | ENSG00000235060 | VDAC1P4 |  |  | 40691 | NaN | 0.3108 | No |
| 66 | ENSG00000234906 | APOC2 |  |  | 41041 | NaN | 0.3050 | No |
| 67 | ENSG00000270858 | VDAC1P5 |  |  | 42711 | NaN | 0.2768 | No |
| 68 | ENSG00000073734 | ABCB11 |  |  | 43160 | NaN | 0.2693 | No |
| 69 | ENSG00000158874 | APOA2 |  |  | 46244 | NaN | 0.2172 | No |
| 70 | ENSG00000232366 | VDAC1P9 |  |  | 46640 | NaN | 0.2105 | No |
| 71 | ENSG00000259948 | AC124068.1 |  |  | 46724 | NaN | 0.2092 | No |
| 72 | ENSG00000234682 | VDAC2P4 |  |  | 48226 | NaN | 0.1839 | No |
| 73 | ENSG00000255776 | VDAC2P2 |  |  | 49680 | NaN | 0.1594 | No |
| 74 | ENSG00000220913 | AL391361.1 |  |  | 51306 | NaN | 0.1319 | No |
| 75 | ENSG00000214976 | VDAC2P1 |  |  | 51939 | NaN | 0.1213 | No |
| 76 | ENSG00000280390 | AC010872.1 |  |  | 52958 | NaN | 0.1041 | No |
| 77 | ENSG00000213262 | VDAC2P3 |  |  | 53267 | NaN | 0.0990 | No |
| 78 | ENSG00000271322 | AL357312.1 |  |  | 53929 | NaN | 0.0879 | No |
| 79 | ENSG00000213450 | VDAC1P7 |  |  | 55723 | NaN | 0.0576 | No |
| 80 | ENSG00000214266 | STARP1 |  |  | 56315 | NaN | 0.0477 | No |
| 81 | ENSG00000132855 | ANGPTL3 |  |  | 57538 | NaN | 0.0270 | No |
Table: GSEA details [plain text format]

  

Fig 2: CHOLESTEROL\_METABOLISM(HSA04979)      
 Blue-Pink O' Gram in the Space of the Analyzed GeneSet

  

Fig 3: CHOLESTEROL\_METABOLISM(HSA04979): Random ES distribution      
 Gene set null distribution of ES for **CHOLESTEROL\_METABOLISM(HSA04979)**
